# Efficacy and safety of Zhenyuan capsule in the treatment of chronic heart failure: A meta-analysis and trial sequential analysis

**DOI:** 10.1097/MD.0000000000035006

**Published:** 2023-09-08

**Authors:** Zengyu Zhang, Jianhe Liu

**Affiliations:** a The First Affiliated Hospital of Hunan University of Chinese Medicine, Changsha, China; b Branch of National Clinical Research Center for Chinese Medicine Cardiology, Changsha, China.

**Keywords:** chronic heart failure, meta-analysis, trial sequential analysis, Zhenyuan capsule

## Abstract

**Background:**

Chronic heart failure (CHF) is a common and difficult-to-treat disease in clinical practice. The efficacy and safety of Zhenyuan capsule (ZYC) in the treatment of CHF were evaluated by meta-analysis and trial sequential analysis (TSA) of published relevant data.

**Methods:**

Searched 8 databases for clinical literature on ZYC in the treatment of CHF, up to December 2022. Then the meta-analysis and TSA were performed on the studies that met the inclusion criteria.

**Results:**

Meta-analysis showed that compared with conventional treatment, combined use of ZYC could significantly increase the clinical effective rate (risk ratio 1.20, 95% confidence interval [CI] 1.14~1.26, *P* < .00001) by 20%, left ventricular ejection fraction (MD 8.85, 95%CI 4.57~13.12, *P* < .0001) by 8.85%, and 6-minutes walking distance (MD 47.91, 95%CI 18.66~77.17, *P* = .001) by 47.91 m, and significantly reduce brain natriuretic peptide (MD −247.86, 95%CI −330.62~−165.09, *P* < .00001) by 247.86 pg/mL. TSA showed that the benefits suggested by the original results were conclusive. In terms of safety, the total adverse events in the combined group of ZYC were comparable to those in the conventional group, and TSA demonstrated that this result needed more research and demonstration.

**Conclusion:**

ZYC can effectively improve the clinical efficacy of treating CHF, significantly increase left ventricular ejection fraction and 6-minute walk distance, and remarkably reduce brain natriuretic peptide. ZYC, with definite efficacy and safety, has the value of clinical application and in-depth research.

## 1. Introduction

Heart failure (HF) is a clinical syndrome caused by structural and/or functional abnormalities of the heart which can result in elevated intracardiac pressure and/or insufficient cardiac output.^[1]^ Patients with HF generally present with dyspnea, ankle edema, and fatigue as the main manifestations, which may be accompanied by signs of jugular venous hypertension, peripheral edema, and lung rupture.^[2]^ HF is clinically divided into acute HF and chronic heart failure (CHF). CHF refers to patients who have been diagnosed with HF or whose symptoms are gradually onset. Epidemiological studies have shown that the incidence of HF in European adults is about 5/1000 per year,^[3]^ and the prevalence of HF in adults is between 1% and 2%.^[4]^ In recent years, the prevalence of HF has trended upward year by year due to the increasing aging of the population.^[5,6]^ It has been reported that the prevalence of HF in people over 70 years of age is up to 10% or more.^[7]^ And, as the population grows and ages, it is expected that the number of hospitalizations for HF may increase substantially in the future.^[6]^ HF is posing a serious threat to human health, significantly reducing the quality of life of patients and contributing to 20% of 1-year mortality and 53% of 5-year mortality from 2000 to 2010.^[8]^ And it is important to note that patients with mildly symptomatic HF still have a higher risk of hospitalization and death.^[9]^ Currently, angiotensin converting enzyme inhibitor (ACEI)/angiotensin receptor-neprilysin inhibitor, β-blockers, and mineralocorticoid receptor antagonist are key agents in the treatment of CHF.^[10]^ They are often combined for the treatment of CHF, and achieved certain clinical efficacy.^[11]^ However, some patients remain uncontrolled in the context of combination drugs, and the long-term combination of these drugs increases the renal burden and gastrointestinal risks.^[12]^ As a result, clinicians are focusing on a number of novel drugs that have the potential to adjuvantly treat CHF and expect them to provide new ideas for the treatment of CHF.

Zhenyuan capsule (ZYC) is a kind of Chinese patent medicine developed by China, the main component of which is the total saponins in fruit of Panax ginseng (SFPG). The theory of Chinese medicine believes that ZYC has the functions of nourishing qi and dredging the veins, calming the mind and tranquilizing the nerves, promoting body fluid and quenching thirst. ZYC was first used for the treatment of angina pectoris in coronary heart disease,^[13]^ and later widely used in various diseases such as coronary heart disease, diabetes, heart failure, and neurasthenia.^[14]^ In 2008, the first study showed that combining ZYC with digitalis, diuretics, and ACEI improved left ventricular ejection fraction (LVEF), cardiac output per minutes, and cardiac index in patients with CHF.^[15]^ Since then, a growing number of studies have shown that ZYC can improve myocardial oxygen metabolism and reduce myocardial oxidative stress injury in patients with CHF through multiple pathways, implying that it may be a therapeutic strategy to consider for CHF.^[16]^ However, there is no meta-analysis or trial sequential analysis (TSA) of ZYC for the treatment of CHF, and the evidence-based evidence for the treatment of CHF with ZYC remains to be elucidated. Thus, this study was conducted to investigate the efficacy and safety of ZYC in the treatment of CHF using conventional drugs as the control group and ZYC combined with conventional drugs as the experimental group.

## 2. Materials and methods

This study strictly followed the systematic review and meta-analysis methodology of the Preferred Reporting Items for Systematic reviews and Meta-Analyses.^[17]^

### 2.1. Literature search

We searched 4 English databases (Embase, the Cochrane Library, Web of Science and PubMed) and 4 Chinese databases (China National Knowledge Infrastructure, VIP, Wanfang and China Biology Medicine). The literature related to ZYC for CHF published before December 2022 was obtained. The subject words covered the terms “ZYC” and “CHF.” The free words were expanded to complete the search in conjunction with the subject words.^[18,19]^

### 2.2. Inclusion and exclusion criteria

We screened the literature for compliance with PICOS principles. Population: met the basic diagnosis of CHF^[20]^; Intervention: the experimental group was treated with ZYCs in combination with the control group; Comparison: The control group was treated with conventional drugs (cardiotonic, diuretics, vasodilators, ACEI, angiotensin receptor-neprilysin inhibitor, β-blockers, etc); Outcome: Clinical effective rate, LVEF, 6-minute walk distance (6MWD), and brain natriuretic peptide (BNP) were used as efficacy endpoints, and total adverse events were used as safety endpoints; Study design: randomized controlled trial.

The exclusion criteria are: Repeatedly published studies; Studies published in abstract form; and Studies for which data are not available.

### 2.3. Literature screening, data statistics and risk of bias

First, in the NoteExpress software, we ruled out inconsistent literature by checking the title, abstract, etc. The remaining literature was reviewed in full text to obtain the final included literature. The next step is to classify and extract the required basic information into statistical tables. Risk of bias was assessed by the assessment tool (Cochrane). The above work was completed independently by 2 authors and checked together, and if there was any disagreement, 3 authors would make a decision.

### 2.4. Statistical analysis

When Revman5.3 was used for meta-analysis, the effect size of the dichotomous variable was established using risk ratio (RR) and 95% confidence interval (CI). The effect size of the continuous variable was calculated using mean difference (MD) and 95% CI. The I^2^ test and Q test were used as standards for heterogeneity analysis. Sensitivity analysis was used to evaluate the stability of the results. TSA 0.9.5.10 Beta software was selected to conduct TSA to determine whether the benefits of the original results were credible. Stata15.0 software was checked for publication bias, which was judged by whether the scatter points were symmetrical and the relationship between the *P* value and .1.

## 3. Results

### 3.1. Literature screening

**Figure 1.**
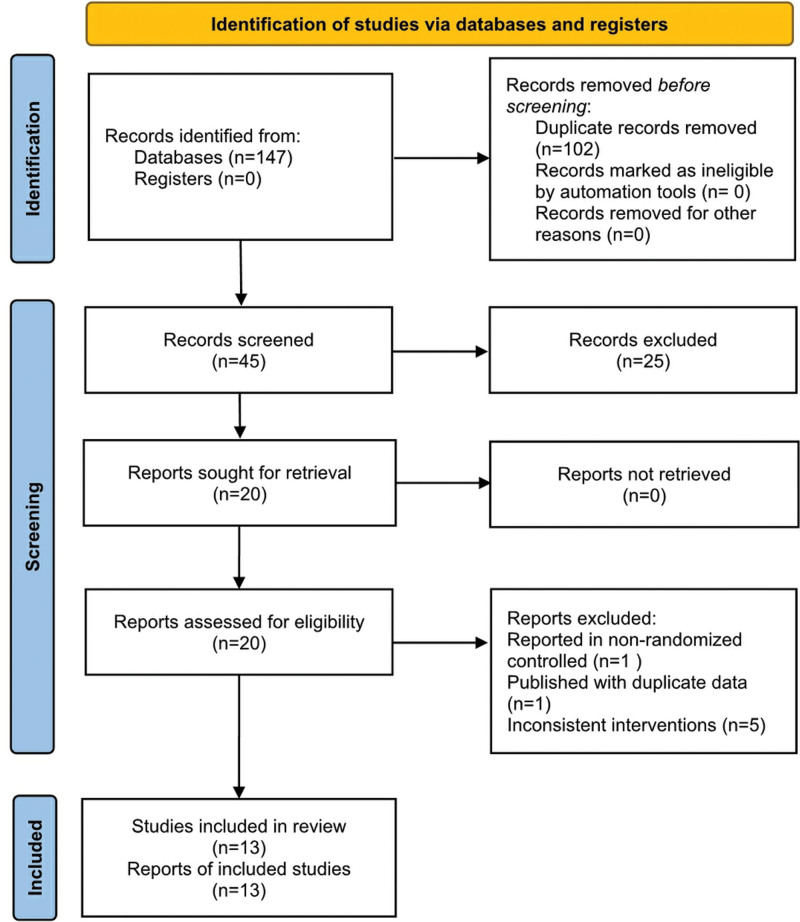
Study flow diagram.

A total of 147 relevant literatures were obtained from the search. Among them, 102 were excluded due to duplication, 25 were eliminated after reading the title and abstract, and 7 were removed after reading the full text, resulting in the inclusion of 13 literature^[21–33]^ (Fig. 1).

### 3.2. Basic characteristics of included studies

A total of 13 clinical trials with a total sample size of 1447 cases were included, and the study centers were all located in China. The basic characteristics of the included studies are shown in Table 1.

**Table 1:** Basic characteristics of the included studies.

Author name	Number randomized (E/C)	Male (%)	Age (Y)	Disease duration (Y)	Intervention	Treatment duration (d)
Cao F 2017	142/142	51.1	37.2	3.2	ZYC 0.5g tid	30
Chen X 2021	30/30	51.7	55.8	/	ZYC 0.5g tid	14
Chen CG 2014	45/44	55.1	61.1	5.3	ZYC 0.5g tid	28
Duan XZ 2014	42/42	58.3	64.4	/	ZYC 0.5g tid	84
Hu JZ 2017	63/63	54.8	65.2	4.3	ZYC 1.0g tid	28
Li DF 2009	60/60	49.2	63.5	4.7	ZYC 1.0g tid	14
Liao YX 2009	39/39	53.8	46.0	6.0	ZYC 0.5g tid	28
Liu ZG 2011	26/26	63.5	/	/	ZYC 0.25g tid	180
Ma S 2015	36/32	57.4	65.8	5.9	ZYC 0.5g tid	28
Wang CL 2020	97/96	48.2	65.6	5.3	ZYC 0.5g tid	84
Wang D 2018	54/54	61.1	57.0	5.5	ZYC 0.5g tid	14
Wen J 2015	53/52	47.6	54.5	/	ZYC 0.5g tid	84
Zhao WP 2019	40/40	50.0	65.3	7.3	ZYC 0.5g tid	30

ZYC = Zhenyuan capsule.

### 3.3. Risk of bias assessment

**Figure 2.**
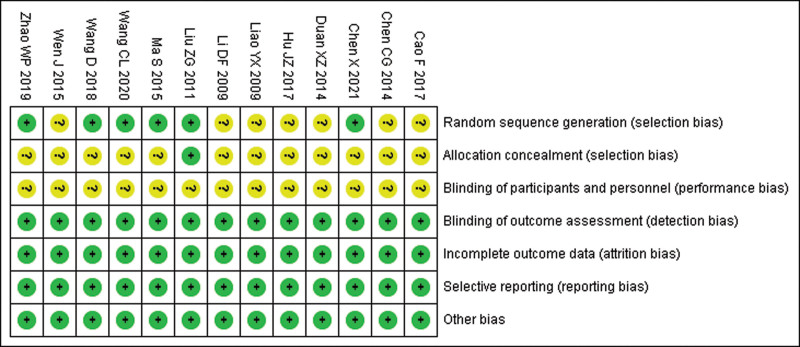
Risk of bias graph.

The risk of bias for randomized methods was unclear in 7 studies. The risk of bias for allocation concealment was unclear in 12 studies. The risk of bias for blinding to patient and participant interventions was unclear in 13 studies. And the risk of bias was low in the remaining domains (Fig. 2).

### 3.4. Efficacy endpoints

#### 3.4.1. Clinical effective rate.

**Figure 3.**
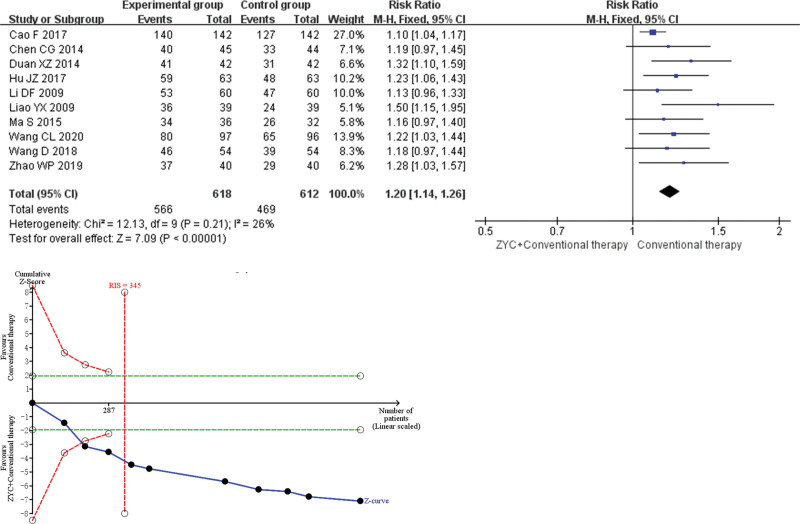
Meta-analysis and trial sequential analysis (TSA) results of Zhenyuan capsule (ZYC) for clinical efficacious rates.

Meta-analysis demonstrated that the combined group of ZYC significantly improved clinical effective rate (RR 1.20, 95% CI 1.14–1.26, *P* < .00001) by approximately 20% compared with conventional therapy. TSA showed a conclusive benefit in clinical effective rate. As shown in Figure 3.

#### 3.4.2. LVEF.

**Figure 4.**
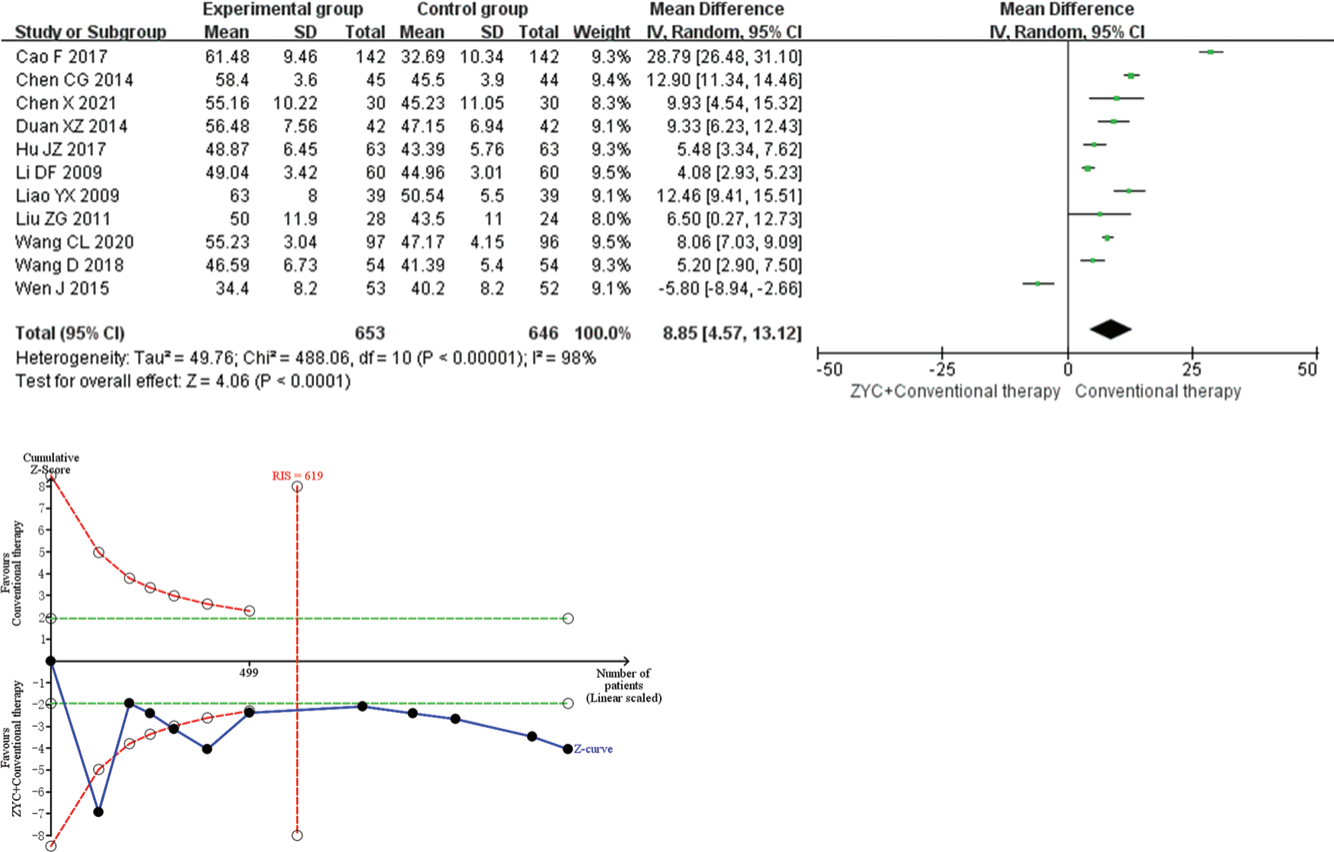
Meta-analysis and trial sequential analysis (TSA) results of Zhenyuan capsule (ZYC) for clinical left ventricular ejection fraction.

Meta-analysis revealed that the combined group of ZYC significantly improved the LVEF (MD 8.85, 95% CI 4.57–13.12, *P* < .0001) by approximately 8.85% compared with conventional therapy. Sensitivity analysis demonstrated low sensitivity and high confidence in the results for combined LVEF. TSA demonstrated that the difference in LVEFF observed in the current information volume was conclusive. As shown in Figure 4.

#### 3.4.3. 6MWD.

**Figure 5.**
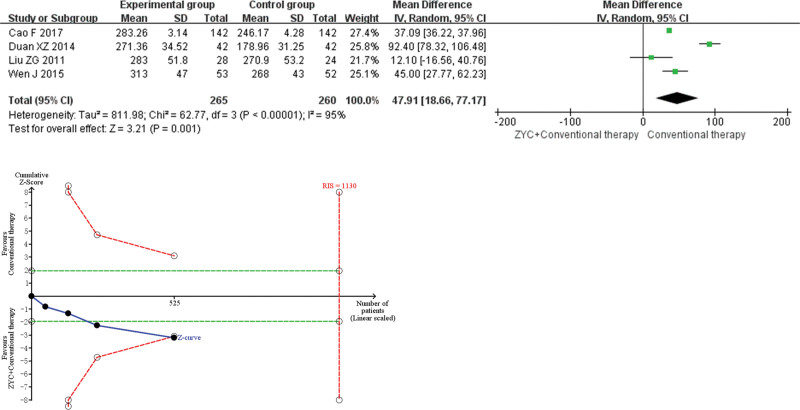
Meta-analysis and trial sequential analysis (TSA) results of Zhenyuan capsule (ZYC) for 6-minute walking distance.

Meta-analysis indicated that the combined group of ZYC significantly improved the 6MWD (MD 47.91, 95% CI 18.66–77.17, *P* = .001) by approximately 47.91 m compared with conventional therapy. Sensitivity analysis displayed heterogeneity in the 6MWD derived from Duan XZ 2014, with no significant change in the combined results after removal of that study (MD 37.09, 95% CI 36.22–37.96, *P* < .00001), suggesting that the result was robust. TSA showed a conclusive benefit for this indicator. As shown in Figure 5.

#### 3.4.4. BNP.

**Figure 6.**
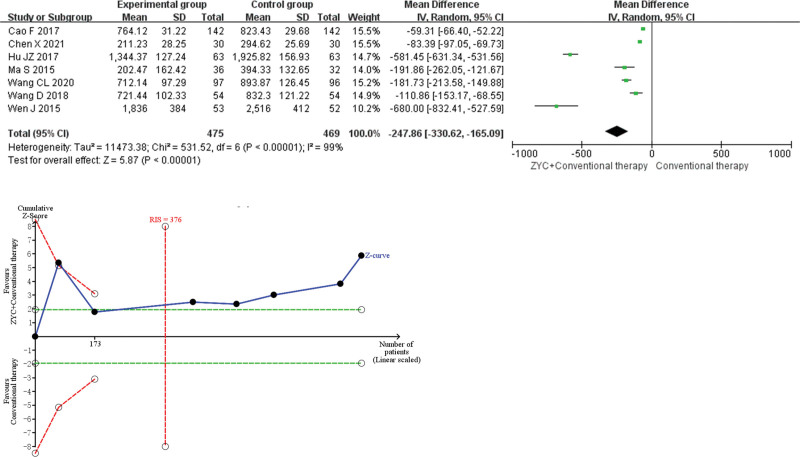
Meta-analysis and trial sequential analysis (TSA) results of Zhenyuan capsule (ZYC) for brain natriuretic peptide.

Meta-analysis demonstrated that the combined group of ZYC significantly reduced BNP (MD −247.86, 95% CI −330.62 to −165.09, *P* < .00001) by approximately 247.86 pg/mL compared with conventional therapy. Sensitivity analysis indicated low sensitivity and high confidence in the results for the combined BNP. TSA displayed the results with exact conclusions for the current amount of information observed. As shown in Figure 6.

### 3.5. Safety endpoint

**Figure 7.**
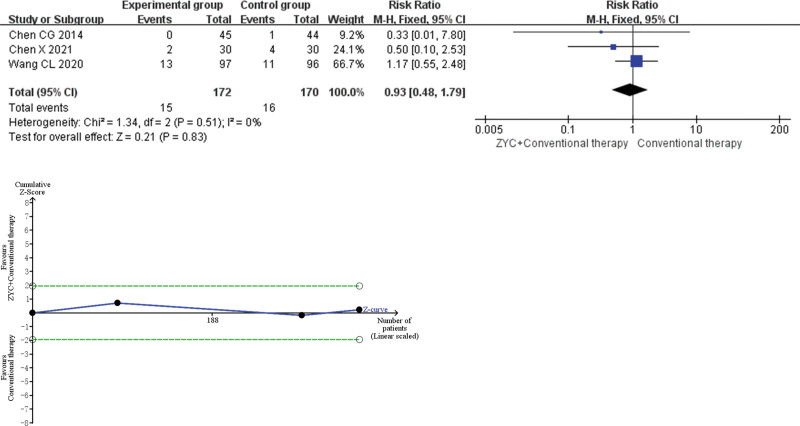
Meta-analysis and trial sequential analysis (TSA) results of Zhenyuan capsule (ZYC) for total adverse events.

Meta-analysis demonstrated that total adverse events in the combined group of ZYC and conventional therapy were comparable (RR 0.93, 95% CI 0.48–1.79, *P* = .83). As shown in Figure 7.

### 3.6. Publication bias

**Figure 8.**
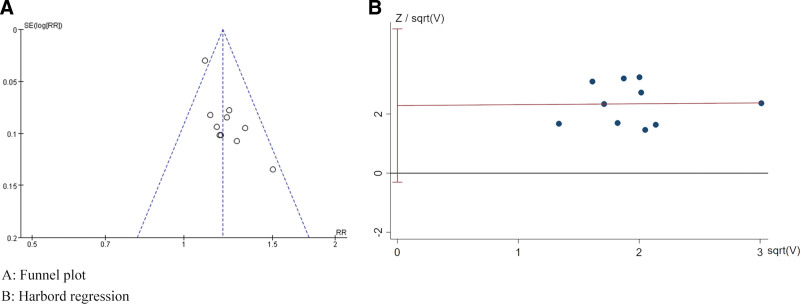
Publication bias assessment.

The inverted funnel plot of clinical effective rates displayed an asymmetric distribution of scatter on both sides and harbord regression showed *P* = .076, suggesting some publication bias in this study. As shown in Figure 8.

## 4. Discussion

CHF is a clinical syndrome caused by multiple causes of cardiac systolic and/or diastolic dysfunction, which leads to limitation of the body activity tolerance and fluid retention.^[34]^ The onset of CHF is mostly related to heart diseases such as hypertension, coronary heart disease, and dilated cardiomyopathy.^[35]^ In addition, damage to vascular endothelial cells can also affect the occurrence and development of CHF.^[36]^ Studies have shown that CHF can reduce vascular shear stress and stimulate the neurohormonal system, leading to a series of oxidative stress reactions and inflammatory reactions, which in turn lead to dysfunction of vascular endothelial function.^[36]^ Although drugs such as angiotensin II receptor/enkephalinase dual inhibitors, β-blockers and sodium-glucose co-transporter 2 inhibitors provide more therapeutic options for the treatment of CHF,^[37]^ mortality in CHF remains at a high level.^[38]^ Therefore, the intervention of novel therapeutic strategies is urgently needed. ZYC is a Chinese patent medicine developed from the SFPG extracted from the ripe fruit of ginseng, which has the functions of nourishing qi and dredging the veins, calming the mind and tranquilizing the nerves, promoting body fluid and quenching thirst.^[39]^ ZYC was first used for the treatment of coronary angina^[13]^ and was first found to improve the prognosis of patients with CHF in 2008. As research progresses, there is increasing evidence which suggests that ZYC are effective in improving cardiac function in patients with CHF and have a good safety profile, which means that it may be a potential drug for the treatment of CHF.^[39]^

In terms of efficacy endpoints, meta-analysis revealed that ZYC improved clinical efficacy by approximately 20%. And TSA indicated that the benefit was conclusive, suggesting that ZYC was effective in improving the overall efficacy of treating CHF. Meta-analysis showed that ZYC significantly increased LVEF by 8.85% and 6MWD by 47.91 m and decreased BNP by 247.86 pg/mL. TSA demonstrated that these benefits were conclusive, implying that ZYC was effective in improving cardiac function in patients with CHF, thereby reducing their clinical symptoms. In terms of safety endpoints, meta-analysis revealed no increase in total adverse events with the combination of ZYC, suggesting that ZYC have a good safety profile. Actually, unlike traditional Chinese medicines with complex ingredients, ZYC is prepared from the Chinese herbal extract SFPG, which may have higher research value due to its clear composition, precise efficacy and high safety.

The mechanism of ZYC for the treatment of CHF may be related to the following 2 aspects: Anti-atherosclerosis. The mechanism of anti-atherosclerosis of ZYC is related to the protection of vascular endothelium, anti-platelet aggregation and anti-inflammation.^[40]^ ZYC can increase nitric oxide levels in blood and reduce endothelin levels, restore the balance of endothelial-platelet-inflammatory regulatory network, slow down the progression of atherosclerosis,^[41]^ and improve myocardial endothelial cell function.^[42]^ SFPG have been reported to promote the release of prostacyclin from vascular endothelial cells and reduce the level of thromboxane A2 in the blood, thereby inhibiting platelet aggregation.^[43]^ In addition, SFPG can inhibit the release of inflammatory factors by suppressing the NF-κB pathway, and thus reduce the inflammatory response of the body; Protection of myocardial cells. SFPG can protect myocardial cells by reducing myocardial oxygen consumption and improving the metabolic level of myocardial cells in order to reduce ischemic necrosis of myocardial cells.^[44]^ Moreover, SFPG improve the ability of endogenous antioxidant enzyme activity by inhibiting lipid peroxidation in myocardial cells and mitigating the damage of myocardial tissue by oxygen free radicals, which effectively improves ventricular structural remodeling and reverses the CHF process.^[44,45]^

Although each step of the analysis in this study strictly followed the relevant guidelines,^[17]^ some limitations still existed. First, 12 clinical trials in this study did not report specific protocols for allocation concealment, and 13 studies did not report blinding of patients and participants to the intervention. These factors increased the risk of selective bias and implementation bias. Second, all the research centers included in the study are located in China, and the research subjects are all Chinese. This means that the benefits of ZYC in the treatment of CHF found in this study may be limited to Chinese, and the role of ZYC in other races is not yet clear. Third, we should pay more attention to the long-term efficacy of drugs in clinical practice because CHF is a disease that requires long-term or even life-long medication. But unfortunately, except for Liu ZG 2011, the treatment courses of the other included studies were all within 3 months. This implies that the results of the study may only reflect the short-term efficacy and safety of ZYC in the treatment of CHF. Fourth, only 3 studies reported drug-related adverse events. TSA showed that the results observed in the current amount of information were not conclusive, so the safety of ZYC needs to be confirmed by more relevant studies. In summary, we expect future research to focus on the use of allocation concealment and blinding of patients and participants to the intervention. We also can pay attention to the efficacy and safety of ZYC in different races, doses and courses of treatment. All these can provide more evidence-based basis for the clinical application of ZYC.

## 5. Conclusion

ZYC can effectively improve the clinical efficacy of treating CHF, significantly increase LVEF and 6MWD, and remarkably reduce BNP. ZYC, with definite efficacy and safety, has the value of clinical application and in-depth research.

## Author contributions

**Conceptualization:** Zengyu Zhang.

**Data curation:** Zengyu Zhang, Jianhe Liu.

**Methodology:** Jianhe Liu.

**Software:** Zengyu Zhang.

**Supervision:** Jianhe Liu.

**Writing – original draft:** Zengyu Zhang.

**Writing – review & editing:** Jianhe Liu.
